# A plant-derived VLP influenza vaccine elicits a balanced immune response even in very old mice with co-morbidities

**DOI:** 10.1371/journal.pone.0210009

**Published:** 2019-01-10

**Authors:** Breanna Hodgins, Stephane Pillet, Nathalie Landry, Brian James Ward

**Affiliations:** 1 Department of Experimental Medicine, McGill University, Montreal, Quebec, Canada; 2 Research Institute of McGill University Health Centre, Montreal, Quebec, Canada; 3 Medicago Inc., Quebec, Quebec, Canada; Icahn School of Medicine at Mount Sinai, UNITED STATES

## Abstract

**Background:**

The elderly are at high risk from influenza, in part because immunity wanes with age and through the accumulation of comorbidities. A novel plant-derived virus-like-particle (VLP) vaccine bearing influenza hemagglutinin can induce a balanced humoral and cellular response in old mice (16–18 months) while split virion vaccines elicit mostly antibodies. Because mice also collect comorbidities and lose immune competence as they age, we wished to determine how the plant-derived VLP vaccine would perform in animals approaching the end of their life-span.

**Materials and methods:**

Old (24–26 months) female BALB/c mice received two intramuscular doses of H1-VLP vaccine, an inactivated H1N1 vaccine (IIV) (both based on A/H1N1/California/07/09) (3μg each) or PBS. Serum was collected on day 42 and humoral responses were measured by enzyme-linked immunosorbent assay (ELISA), microneutralization (MN) and hemagglutination inhibition (HI) assays. Influenza-specific splenocyte CD4^+^ & CD8^+^ T cell responses were measured by flow cytometry. Full body computed tomography (CT) and structured necropsies were performed on day 42. Comorbidities including reduced lung volume (kyphosis), masses, abscesses, etc. were assessed using a standard scoring system (1–21) and mice with scores ≥5 were considered to have important comorbidities.

**Results:**

Overall, 53.3% of the animals had significant comorbidities. Three weeks post-boost, HI and MN titres were mostly undetectable but ELISA titres were significantly higher in the H1-VLP animals compared to the IIV group (GMT (95% CI): 961 (427, 2163) vs 425 (200, 903): p = 0.03). Both CD4^+^(TNFα, IFNγ) and CD8^+^ (IFNγ) T cell responses were also greater in the H1-VLP group than the IIV.

**Conclusions:**

Even in very old mice with comorbidities, the plant-made H1-VLP vaccine elicited a stronger and more balanced immune response than IIV. Animals with fewer comorbidities tended to have the better composite (humoral and cellular) responses. These novel vaccines have the potential to address some of the limitations of current vaccines in the elderly.

## Introduction

The health risks of influenza increase steadily with age such that >70% of the mortality associated with seasonal outbreaks occurs in those ≥ 65 years of age [1]. Unfortunately, the efficacy of current split-virion influenza vaccines typically diminishes as people grow older. Even with vaccines that have been designed, at least to some extent, for this population (eg: high dose, MF59-adjuvanted), vaccine efficacy (VE) can still be very low [2]. One factor that likely contributes to poor VE in older subjects is the fact that the vaccines used in adults and the elderly have all been optimized for antibody production, and specifically antibodies that react in the hemagglutination inhibition (HI) assay. In recent years, there has been increasing evidence that older individuals are protected from influenza primarily by cellular rather than antibody responses [3–5]. Although protection is far from optimal, the elderly can derive benefit from seasonal influenza vaccination despite making little-to-no antibody response [6–8]. Other factors that contribute to low VE in the elderly are immunosenescence, a gradual weakening in multiple components of the immune system, and the accumulation of chronic inflammatory and comorbidities conditions with age (eg: arthritis, diabetes, cancer, degenerative diseases, etc.) (so-called ‘inflamm-aging’) [9, 10]. There is a clear need to develop influenza vaccines that will provide better protection for the growing elderly population of the world.

Virus-like particles (VLPs) have features that theoretically make them attractive as vaccine candidates across all ages. These include rapid transport to lymphatic tissues, delivery of an antigen bolus to antigen-presenting cells (APC) and activation of APCs leading to stimulation of both humoral and cellular responses [11–14]. We have recently demonstrated that many of these theoretical advantages can be realized with plant-made VLPs bearing the hemagglutinin proteins (HA) of pandemic and seasonal influenza viruses [15]. In both pre-clinical studies with young animals and a series of clinical trials in healthy young adults, we have shown that these VLPs not only elicit strong and cross-reactive antibody responses but also induce long-lived and poly-functional CD4^+^ T cell responses [14, 15]. Based on these results, an obvious question to ask was whether or not some of these same advantages would also be seen at older ages. Like the elderly, old mice also suffer from chronic inflammatory conditions and comorbidities, such as cancers, abscesses, rectal or uterine prolapse, skin lesions, renal disease, etc. [16]. In this work, we compared the humoral and cellular responses to a candidate H1-VLP vaccine (H1N1 A/California/07/2009) to those seen with a split vaccine in very old mice with natural comorbidities.

## Materials and methods

### Virus, mice and vaccines

Female BALB/c mice (Charles River Laboratories, Montreal, QC) near the end of their life-spans (24–26 months age) were vaccinated with two doses of phosphate buffered saline (PBS), H1-VLP vaccine (Medicago, Quebec City, QC) or split inactivated H1N1 vaccine (BEI resources, Manassas, VA). The latter two were based on H1N1 A/California/07/2009 (pdmH1N1)– 3 μg /dose based on HA content in 50 μL for the H1-VLP and 100 μL for the split vaccines. Vaccines were administered intramuscularly (IM) 3 weeks apart (d0 and d21) and animals were sacrificed on d42. The H1-VLP was produced by transient expression in *Nicotiana benthamiana* plants as previously described [17] using the wild-type HA sequence from pdmH1N1. The H1-VLP or PBS were administered into the quadriceps muscle using a 28G ½ needle. The split vaccine was administered to both legs into the quadriceps muscle. The study included 9–17 mice per group.

Peripheral blood was collected in microtainer serum separator tubes (BD Biosciences, Mississauga, ON) from the lateral saphenous vein before immunization (d0), and at d21 post-vaccination. Sera were obtained using the manufacturer’s instructions and stored at -20°C. At d42, mice were sacrificed by isoflurane followed by a CO_2_ chamber. Serum was collected by cardiac puncture and splenocytes were isolated as previously described [18]. All procedures were carried out in accordance with guidelines of the Canadian Council on Animal Care, as approved by the Animal Care Committee of McGill University.

### Generation of H1-VLPs

H1-VLP were generated as previously described [13, 17, 19]. Briefly, *Nicotiana benthamiana* plants were vacuum infiltrated in batches with an *Agrobacterium* inoculum containing an H1 expression cassette. Approximately a week after infiltration, the plants were harvested, homogenized and undergo several purification steps. The HA content for the H1-VLP was assessed by single radial immunodiffusion (SRID) assay.

### Antibody titer measurements

The hemagglutination inhibition (HAI) [13] and microneutralization (MN) [20] were performed essentially as previously described. Briefly HAI were performed as follows: mouse sera and receptor-destroying enzyme (RDE: Cedarlane, Burlington, ON) were mixed 1:4 and incubated. The RDE was inactivated and sera were serially diluted in phosphate buffered saline (PBS: pH: 7.4) in 96-well V-bottom plates (Coring Inc Costar, Corning, NY). Diluted sera were then incubated with 8 HA units of pdmH1N1 before 0.5% turkey erythrocytes diluted in PBS (50 μL /well: Lampire biological Laboratories, Pipersville, PA) were added to wells. The HAI titer was determined by visual inspection as the highest dilution that inhibited erythrocyte agglutination using standard criteria. MNs were as follows: confluent monolayers of Madin-Darby canine kidney (MDCK: British Colombia Center for Disease Control) cells were incubated in 96 well, flat-bottom plates (Falcon Corning Life Science, Corning, NY) in supplemented MegaVir Sera were heat-inactivated and diluted 2-fold in MegaVir in duplicate wells. Each well then received 100 infectious units of pdmH1N1 diluted in MegaVir. Cytopathic effect (CPE) was assessed at 4 days and the titer was defined as the reciprocal of the highest serum dilution to completely block CPE. Both HAI and MN assays used H1N1 A/California/07/2009 (National Microbiology Laboratory, Public Health Agency of Canada).

Enzyme-linked immunosorbent (ELISA) assays were performed as previously described [18]. Briefly, high-binding 96-well plates (Greiner Bio-one, Frickenhausen, Germany) were coated overnight at 4°C with recombinant HA from pdmH1N1 (Immune Technologies, New York, NY) (0.5 μ μg/mL). Each plate contained a standard curve with 2-fold dilutions of purified mouse IgG (Sigma, St. Louis, MO) starting at 2000 ng/mL. Wells were blocked with 2% bovine serum albumin (BSA; Sigma) in PBS-Tween 20 (0.05%; Fisher Scientific, Ottawa, ON). Sera were heat inactivated, diluted 1:50 in blocking buffer and added in duplicates (50 μL/well). Plates were incubated for 1 hour at 37°C. HRP-conjugated anti-mouse total IgG antibodies (Jackson ImmunoResearch Laboratories Inc., West Grove, PA) diluted 1:50,000 in blocking buffer was added (75 μL/well, 0.5 hr at 37°C). 3,3’ 5,5’-tetramethyl benzidine (TMB) substrate (100 μL /well: Millipore, Billerica, MA) was used for detection followed by 0.5 M of H_2_SO_4_ after 15 minutes (50 μL /well). Optical density (OD) was measured at 450 nm with an EL800 microplate reader (BioTek Instruments Inc., Winooski, VT). The concentration of HA-specific IgG was calculated using the mouse IgG standard curve.

### Splenocyte isolation and stimulation

Individual spleens were harvested at d42 into Hank’s balanced salt solution at room temperature (RT) without calcium or magnesium (HBSS -/-) (Wisent, St. Bruno, QC), and processed as previously described [18]. Fresh splenocytes were seeded in 96-well U-bottom plates (BD Falcon, Mississauga, ON) in 200 μL per well (1x10^6^ cells/well). Duplicate cultures were stimulated with cRPMI alone (unstimulated), H1-VLP (2.5 μg/mL HA) or PMA+ ionomycin (each 1 mg/mL) (Sigma, St. Louis, MO) for 18 hours at 37°C at 5% CO_2_. After 13 hours, protein transport inhibitor was prepared according to manufacturer’s instructions (BD Science, San Jose, CA) and added to samples (20 μL/well). Five hours later, plates were spun (320xg, 8 minutes at 4°C) and cells were processed for flow cytometry as described below. In our hands, we see no significant differences in T cell responses after splenocyte re-stimulation *ex vivo* with inactivated virions, VLPs or recombinant HA.

### Flow cytometry

Splenocytes were washed twice with 200 μL of cold PBS (pH: 7.4, Wisent) then centrifuged at 320xg for 8 minutes at 4°C. Viability dye (50 μL/well diluted at 1:100) (Affymetrix ebioscience, Waltham, MA) was added and incubated for 20 minutes at 4°C in the dark. Cells were washed as above and Fc block (1μL/well, BD Science, San Jose, CA) was added. Plates were incubated for 20 minutes at 4°C. For the surface stains, each antibody was diluted at 1:100 in PBS and 50 μL/well of the extracellular ‘cocktail’ was added for 30 minutes at 4°C protected from light. The following antibodies were used: CD3 –FITC (Clone:145-2C11, Affymetrix ebioscience), CD4-V500 (RM4-5, BD Bioscience) and CD8-PerCP-Cy5 (Clone:53–6.7, BD Bioscience). Cells were washed as above, then 100 μL/well of fixative was added (BD Science) for 30 minutes or overnight at 4°C protected from light. For intracellular staining, cells were washed as above with 1X permeabilization buffer (BD Science) then stained with an intracellular ‘cocktail’ of antibodies as 1:50 dilution in permeabilization buffer (50 μL/well). The following markers were used: IL-2-Pe-Cy5 (Clone: JES6-5H4, Biolegend, San Diego, CA), IFNγ-PE (Clone: XMG1.2, BD Science) and TNFα-efluor450 (Clone:MP6-XT22, Affymetrix ebioscience) and incubated for 40 minutes in the dark at 4°C. After washing with PBS, cells were fixed with intracellular fixative (Affymetrix ebioscience) and analyzed on BD LSRFortessa X-20 (BD Science) using Flowjo software (version 10.0.8r1). Our gating strategy is shown in S1 Fig.

### Comorbidity assessment

Immediately prior to sacrifice, comorbidities were assessed by full body CT scans (Mediso nanoScan SPECT/CT/PET, Budapest, Hungary) and each major organ was evaluated as either normal (score 0), slightly abnormal (score 1) or grossly abnormal (score 2). At the time of sacrifice, a structured necropsy was performed and each major organ was visually assessed and scored (as above). An overall score for each animal was calculated (maximum score 18).

### Computed tomography

At d42, full body CT scans were performed 5 minutes after tail-vein injection of a contrast agent (Omnipaque: GE Healthcare, Mississauga, ON). These images were analyzed with Amide software (version 1.0.4: sourceforge.net). Lung volumes were estimated by measuring the distance from the lung apices to diaphragm (vertical) on the coronal view and from the spine to the sternum (horizontal) on the sagittal view (population terciles scored 1–3). CT images were blinded for analysis. CT scans scores and necropsy scores were combined for a total score.

### Statistical analysis

For statistical analysis, one-way ANOVA or two-way ANOVA tests were performed on the log10 values for sera analysis. For statistical analysis, one-way ANOVA was used on the log values for A). For B) Mann-Whitney test was performed on the log values. Values lower than the detection limit for the ELISA assays were adjusted to 2x the GMT of the PBS group since GMT values cannot be calculated with values equal to zero. Two-way ANOVA test was used for cytokine data. All analyses were performed using GraphPad Prism 6.0 software. Outliers were identified and removed by ROUT using GraphPad Prism.

## Results

### Humoral response

**Fig 1:**
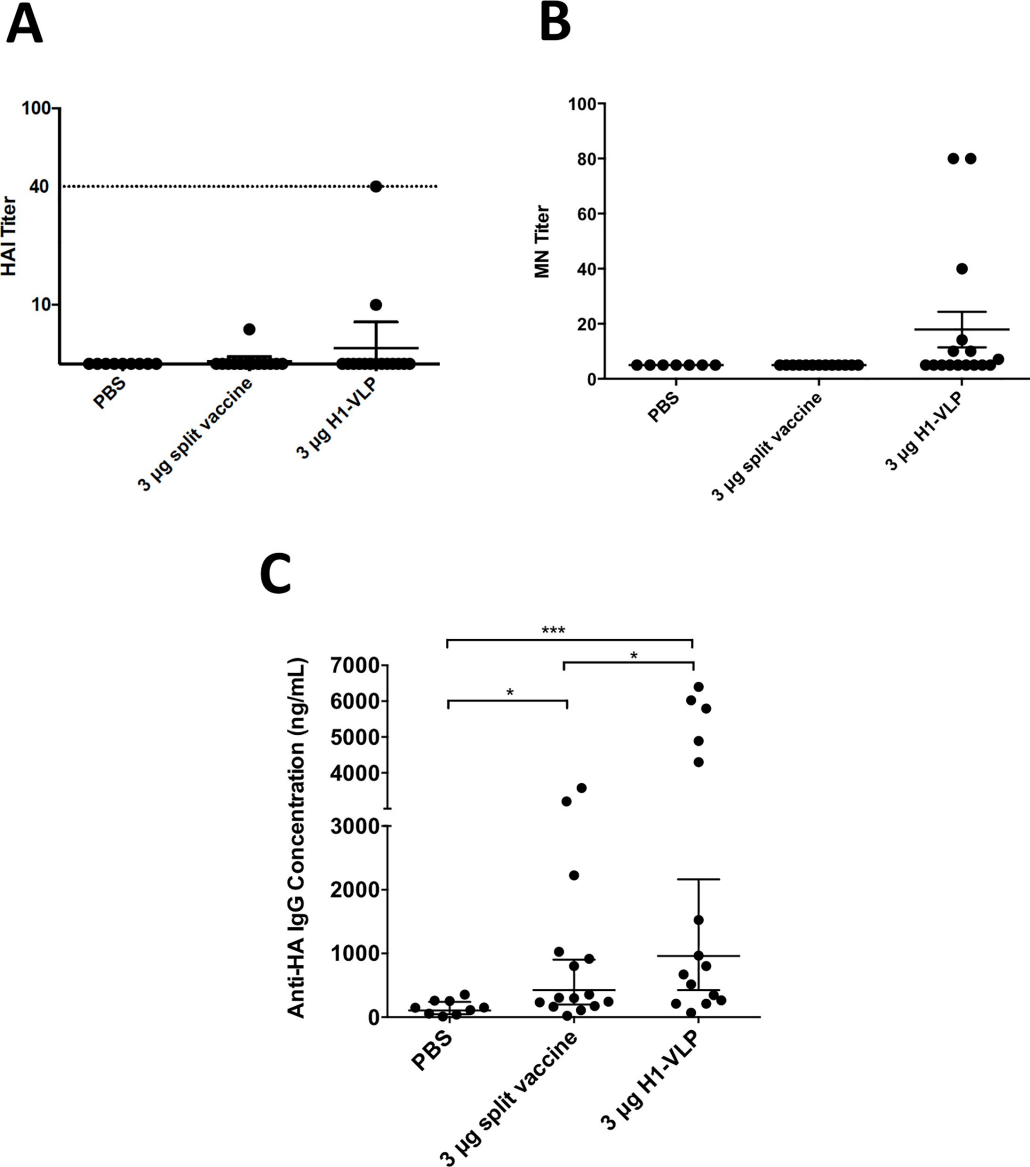
Serum antibody response after two doses of H1-VLP or split-virion vaccine. Aged (24–26 months) BALB/c mice were immunized twice with H1-VLP, split-virion vaccine or PBS. Three weeks post-boost sera from individual mice were analyzed by Hemagglutination Inhibition (HI) (A) and microneutralization (MN) (B) titer against A/California/07/2009 H1N1. Influenza HA-specific IgG concentrations (C) by ELISA. Dotted line in A) represents 40 HAI which is considered the protection level in humans. For statistical analysis, one-way ANOVA was used on the log values for A). For B) and C) Mann-Whitney test was performed on the log values (*** p<0.001, * p<0.05). A), B) and C) represent 9–15 mice/group.

Not unlike older humans, these very old mice had undetectable or limited humoral responses to vaccination using the standard influenza vaccine serologies (HI and MN, Fig 1A & 1B respectively). Only one or two mice in each vaccine group mounted any detectable HI response (GMT: 40 and 10 or 7.1 in the H1-VLP or IIV groups respectively). All other mice had HI titres below the limit of detection of our assay (≤10). No animal in the PBS or IIV groups mounted a detectable MN response. In contrast, 7/15 (46.7%) of the mice in the H1-VLP group had detectable MN responses although the overall MN response remained low (GMT:10: range 10–80). Antibodies measured by ELISA (Fig 1C) were detected in both active vaccine groups. If 2x the GMT of the PBS group is defined as the lower limit of detection in this assay, 11/15 (73.3%) of the IIV group and 12/15 (80.0%) of the H1-VLP group mounted detectable responses. The overall GMT in the H1-VLP groups was 2 times higher than the IIV group (961 versus 425: p = 0.03).

### CD4^+^ and CD8^+^ T Cell responses

**Fig 2:**
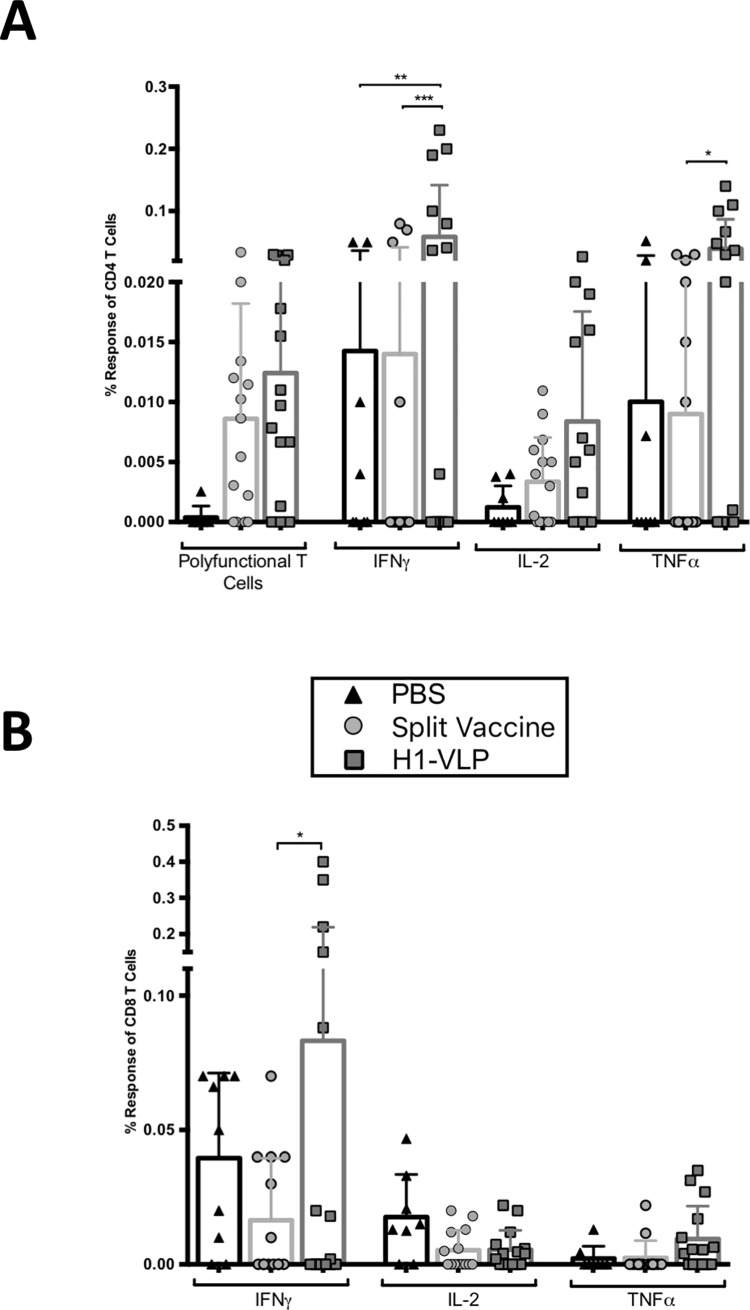
Cytokine production by splenocytes after *ex vivo* stimulation three weeks post-boost. Aged (24–26 months) BALB/c mice were immunized twice with H1-VLP, split-virion vaccine or PBS. Three weeks post-boost, splenocytes were collected and stimulated *ex vivo* for 18 hours with H1-VLP. Flow cytometry was performed to determine the following cell types and cytokine expression: A) CD4^+^ T cells and B) CD8^+^ T cells. Subtractive data was used (Stimulated—unstimulated). For statistical analysis, two-way ANOVA was performed (**** p<0.0001, *** p<0.001, ** p<0.01, * p<0.05). Data represent 9–15 mice/group.

The PBS group had overall low T cell production of IFNγ, TNFα and IL-2. The CD4^+^ and CD8^+^ T cell responses following re-stimulation with H1 antigen *ex vivo* tended to be higher in the H1-VLP group than the IIV animals (Fig 2). Differences between the H1-VLP and IIV groups reached statistical significance for CD4^+^ T cells expressing IFNγ and TNFα (Fig 2A) and for CD8^+^ splenocytes expressing IFNγ (Fig 2B). In the H1-VLP group, there was a higher percent of poly-functional CD4^+^ T cells observed, compared to than the IIV group (0.012 vs 0.009, respectively) but this difference did not reach statistical significance (Fig 2A). Although these percentages are small, the number of cells that they represent for this specific antigen are substantial.

### Comorbidities

**Fig 3:**
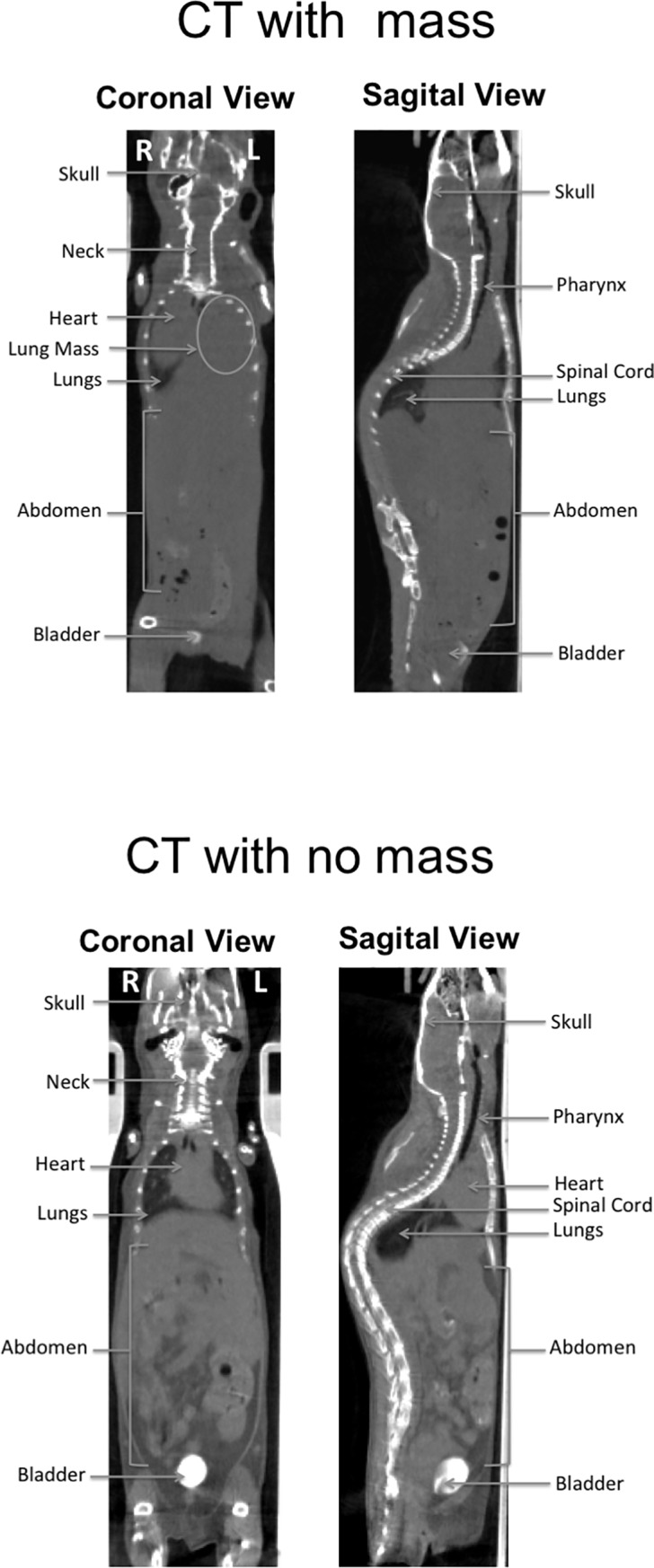
Full body CT scans three weeks post-boost vaccination. Aged (24–26 months) BALB/c mice were immunized twice with H1-VLP, split-virion vaccine or naïve. Three weeks post-boost, 4–15 mice/group underwent full body CT scans. A) Depicts a mouse with the presence of mass on the left side of its chest. B) demonstrates a relatively healthy aged mouse with no mass detected.

Mice in all of the groups had obvious but varied comorbidities; enlarged and/or granular-appearing spleens, pale spleens (anemic), liver nodules, or abscesses, enlarged heart, inflammation/abscesses in ovaries and uterus, skin and tail erosions/ulcers. Masses (presumed tumors) were found in the liver, neck, lungs or uterus/ovaries of some animals (Fig 3). The abnormalities tended to be evenly distributed between the groups as demonstrated by the total comorbidity scores (Table 1). Overall comorbidity scores were approximately the same between the H1-VLP group and the split-inactivated virion (5.33 ± 1.72 and 5.00 ± 2.54, respectively) and 53.3% (8/15) of mice in each group had at least 1 readily identified comorbidity. There was no overall correlation between morbidity scores and either antibody or cellular responses.

**Table 1:** Summary of comorbidity scores assessed by necropsy and CT scan after two immunizations of H1-VLP, split-virion vaccine or PBS.

	Lung Volume Score	Necropsy Score	Total
**PBS**	2.25 ± 0.96	3.25 ± 1.89	5.5 ± 1.73
**3 μg split vaccine**	2.47 ± 0.92	2.53 ± 2.03	5.00 ± 2.54
**3 μg H1-VLP**	2.47 ± 0.83	2.87 ± 1.60	5.33 ± 1.72

## Discussion

The aging population is one of the major risk groups for influenza morbidity and mortality. Unfortunately, the currently available vaccines that target the elderly are far from optimal in the protection they provide. The development of vaccines that can stimulate cellular responses in addition to antibodies may be of particular interest for this population since older individuals appear to derive significant benefit from cellular memory [3, 21]. We have recently demonstrated that VLP vaccines made in plants that carry HA trimers of either seasonal or pandemic influenza viruses can protect animals (mice, ferrets) in lethal challenge studies despite low and, in some cases, even completely absent antibody responses [22, 23]. After footpad injection in a mouse vaccination model, these VLPs have been shown to move to regional lymph nodes (LN) within minutes where they preferentially associate with antigen-presenting cells (APC) and B cells [24] linked with a rapid increase in LN cellularity and dendritic cell activation [25]. The *in vitro* interactions of these VLPs with human immune cells are also quite unusual. VLPs bearing HA trimers of seasonal strains cause rapid clustering of peripheral blood mononuclear cells (PBMC) leading to expression of activation markers and the release of pro-inflammatory cytokines by monocytes [26]. In a process mediated by cell-surface sialic acid residues, HA-bearing VLPs are rapidly internalized by human APCs including monocyte-derived macrophages [27] and monocyte-derived dendritic cells (unpublished data). These animal and *in vitro* observations suggest that the plant-derived VLPs interact with immune cells very differently from inactivated split-virion vaccines [26, 27].

In clinical trials conducted to date, VLPs have been shown to elicit a more balanced humoral and cellular response than the Th2 (antibody)-dominated response typically seen with IIV comparators in healthy adults and older subjects (≥65 years of age) ([15], unpublished data). In all of these trials however, subjects with serious comorbid conditions were excluded. These clinical observations are consistent with previous studies showing that healthy BALB/c mice up to 16–20 months of age can be protected from lethal challenge with a single dose of the plant-derived VLP vaccine despite relatively weak (younger animals) or absent (older animals) antibody responses [22]. In the current work, we extended our mouse model to the limit of the murine life-span. Under laboratory conditions, BALB/c mice are considered to be very old when they reach 18–20 months of age [28, 29]. Even though BALB/c mice accumulate age-related pathologies at a slower than other in-bred strains [30], more than half (53.3%) of the animals in our study had at least 1 obvious comorbid condition identified by either whole body CT or structured necropsy at study termination. Although we observed a robust ELISA response in these very old mice after two doses of the VLP vaccine that was clearly superior to that seen in IIV recipients, even the VLP-vaccinated animals made almost no detectable HI or MN antibodies. However, the most striking differences between VLP- and IIV-treated animals were seen in the T cell responses. Based on antigen-specific intracellular cytokine responses, the VLP vaccinated animals had readily-detectable CD4^+^ (ie: poly-functional and individual cytokines) and CD8^+^ (ie: IFNγ) T cell responses that were clearly superior to those seen in the IIV and PBS control groups. Although not enough very old animals were available to perform a terminal challenge in this study, these data nonetheless suggest that the VLP-vaccinated animals would likely have been better-protected than the IIV recipients’ due to the superior immunogenicity of the plant-derived VLPs. There are several methods in producing VLPs other than the plant system, such as my mammalian or insect cell lines. All of these systems also differ in their glycosylation patterns.

Although influenza vaccine licensing strategies have long placed a heavy emphasis on a new product’s ability to elicit high HAI titers [31], older adults typically make much lower HAI and neutralizing antibody responses than younger adults [6] and there is a clear inverse correlation between health status and the HAI response. Older adults with multiple co-morbidities have even lower antibody responses to IIV than the healthy elderly [32]. Although a robust humoral response to influenza virus antigens can undoubtedly help to prevent infection, there is a growing appreciation that other factors need to be considered in assessing influenza vaccine-induced immunity including antibodies than those measured in the classical HI and MN assays (eg: anti-stalk) [33] and cellular immunity including NK and T cell responses [34]. Such considerations may be particularly important in designing and evaluating new influenza vaccines for the elderly [3].

Our work has several obvious limitations, the most important of which were the relatively small numbers of very old animals available for study (ie: no challenge to directly assess efficacy) and the fact that morbidity evaluation in mice is very far from an exact science. Although we tried to standardize our comorbidity assessment by using both whole body CT and a structured autopsy, these very old animals were so fragile that substantial mortality occurred in performing the one CT examination during a pilot study (25%). Therefore we did not want to risk losing mice with serial imaging and made extensive handling impossible (ie: frailty assessment) [35]. Furthermore, although some of the parameters we measured have obvious parallels in the elderly (ie: kyphosis and restricted lung volume) [36], we cannot claim that the spectrum of comorbidities observed in the very old mice are an accurate reflection of those experienced by aging humans. Finally, although the overall co-morbidity ‘score’ was similar between groups in our study, we could not ensure that there was an equal distribution of the different kinds of comorbidities across groups.

Although sufficient numbers of animals were not available in the current study to perform a challenge, our previous work with healthy old mice [22] strongly suggests that the pattern of immune response elicited by the VLP vaccine should provide better protection than IIV even in very old mice with multiple co-morbidities. These data also suggest that guarded optimism is appropriate for the outcome of an on-going efficacy study in subjects ≥65 years of age comparing the plant-derived quadrivalent VLP vaccine to a commercial comparator. Such an outcome would be highly desirable since the elderly are currently not well-served by the available vaccines against influenza.

## Supporting information

S1 Fig Flow cytometry-gating strategy for splenocytes. Aged (24–26 months) BALB/c mice were immunized twice with H1-VLP, split-virion vaccine or naïve. Three weeks post-boost (9–15 mice/group), splenocytes were collected and stimulated *ex vivo* for 18 hours with H1-VLP. This strategy was done for each cell type CD4 and CD8 T cells and for each of those cell types the following cytokine expression was calculated: IFNγ, TNFα and IL-2. Subtractive data was used (Stimulated—unstimulated).
(TIFF)
