# Supplementary figures and images for: A plant-derived VLP influenza vaccine elicits a balanced immune response even in very old mice with co-morbidities
Source: PLoS One. 2019 Jan 10;14(1):e0210009. doi: 10.1371/journal.pone.0210009 (PMC6328122; doi:10.1371/journal.pone.0210009)

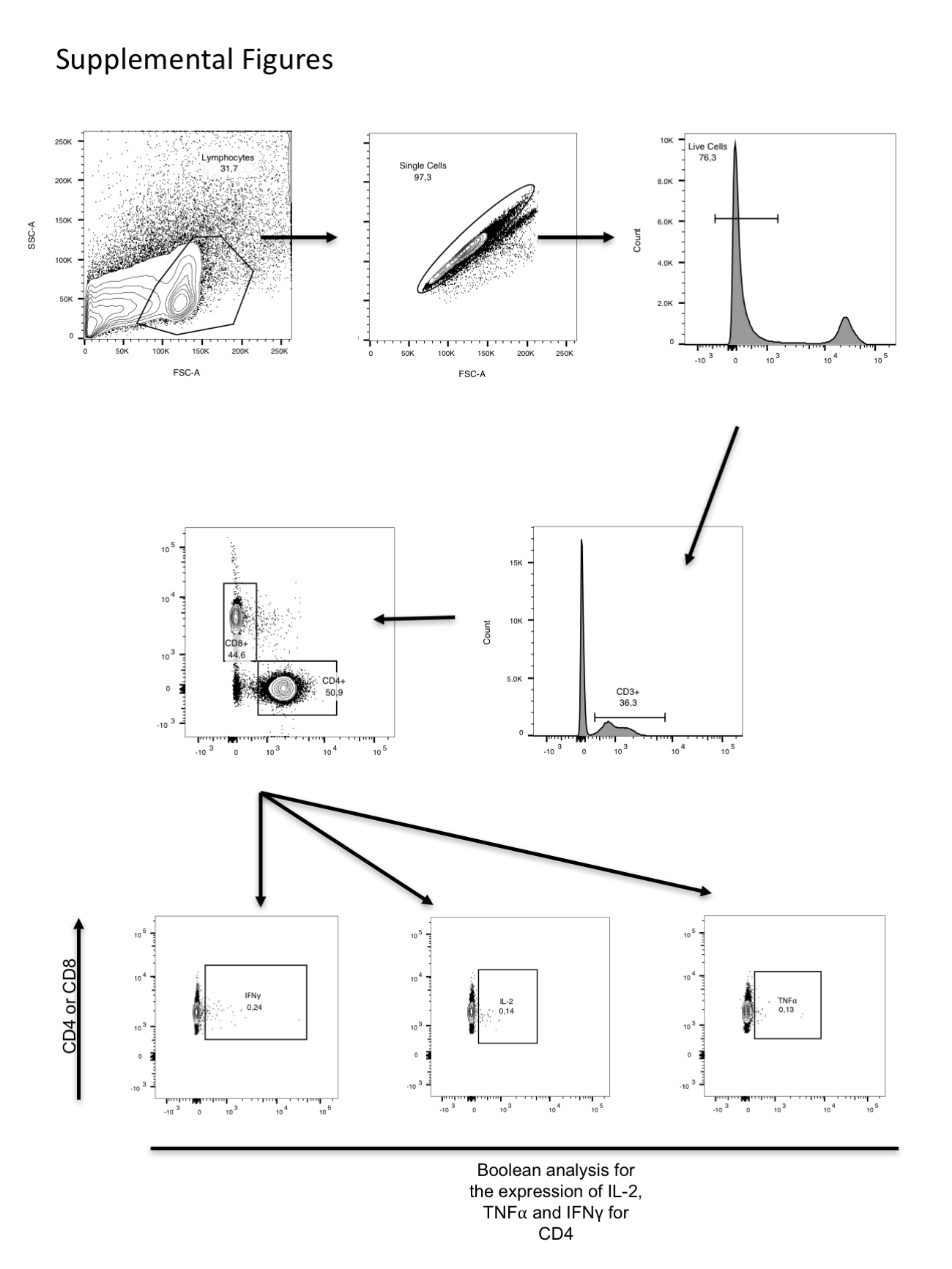

Supplement: S1 Fig — Aged (24–26 months) BALB/c mice were immunized twice with H1-VLP, split-virion vaccine or naïve. Three weeks post-boost (9–15 mice/group), splenocytes were collected and stimulated ex vivo for 18 hours with H1-VLP. This strategy was done for each cell type CD4 and CD8 T cells and for each of those cell types the following cytokine expression was calculated: IFNγ, TNFα and IL-2. Subtractive data was used (Stimulated—unstimulated). (TIFF) [file pone.0210009.s001.tiff]
